# Trans-oligomerization of duplicated aminoacyl-tRNA synthetases maintains genetic code fidelity under stress

**DOI:** 10.1093/nar/gkv1020

**Published:** 2015-10-12

**Authors:** Miguel Ángel Rubio, Mauro Napolitano, Jesús A. G. Ochoa de Alda, Javier Santamaría-Gómez, Carl J. Patterson, Andrew W. Foster, Roque Bru-Martínez, Nigel J. Robinson, Ignacio Luque

**Affiliations:** 1 Instituto de Bioquímica Vegetal y Fotosíntesis, C.S.I.C. and Universidad de Sevilla, Avda Américo Vespucio 49, E-41092 Seville, Spain; 2 Facultad de Formación del Profesorado. Universidad de Extremadura, Avda de la Universidad s/n. E-10003, Cáceres, Spain; 3 SBBS and Chemistry, Durham University, Durham DH1 BLE, UK; 4 Department of Agrochemistry and Biochemistry, Faculty of Science, University of Alicante, E-03080, Spain

## Abstract

Aminoacyl-tRNA synthetases (aaRSs) play a key role in deciphering the genetic message by producing charged tRNAs and are equipped with proofreading mechanisms to ensure correct pairing of tRNAs with their cognate amino acid. Duplicated aaRSs are very frequent in Nature, with 25,913 cases observed in 26,837 genomes. The oligomeric nature of many aaRSs raises the question of how the functioning and oligomerization of duplicated enzymes is organized. We characterized this issue in a model prokaryotic organism that expresses two different threonyl-tRNA synthetases, responsible for Thr-tRNA^Thr^ synthesis: one accurate and constitutively expressed (T1) and another (T2) with impaired proofreading activity that also generates mischarged Ser-tRNA^Thr^. Low zinc promotes dissociation of dimeric T1 into monomers deprived of aminoacylation activity and simultaneous induction of T2, which is active for aminoacylation under low zinc. T2 either forms homodimers or heterodimerizes with T1 subunits that provide essential proofreading activity *in trans*. These findings evidence that in organisms with duplicated genes, cells can orchestrate the assemblage of aaRSs oligomers that meet the necessities of the cell in each situation. We propose that controlled oligomerization of duplicated aaRSs is an adaptive mechanism that can potentially be expanded to the plethora of organisms with duplicated oligomeric aaRSs.

## INTRODUCTION

Functional innovation rarely occurs by the *de novo* tailoring of sequences but most frequently by co-option of pre-existing functional domains or full-length polypeptides. Divergence of duplicated genes is thought to be a major force in evolution (1). Though in most cases, one of the gene copies degenerates and disappears, it may happen that both copies are fixed in the population by positive natural selection or genetic drift. Once fixed, genes can evolve in distinct ways that may lead to the adoption of novel functions. Duplicated essential genes may also evolve asymmetrically provided that the original function is maintained, either by one of the copies or by the joint action of both genes (2). The latter case often requires the parallel evolvement of regulatory systems to coordinate the action of the two copies. For genes encoding modular proteins, evolution may operate distinctly on the different domains. Therefore, the evolution of duplicated genes encoding modular proteins may be complex, with domains evolving with relative independence to other domains *in cis* and *in trans* (1). Deciphering the functional role of duplicated genes after divergence is rarely straightforward and often requires dedicated experimental approaches.

Gene duplication is thought to have played a major role in the evolution of aminoacyl-tRNA synthetases (aaRSs), a family of essential enzymes that provide the aminoacyl-tRNAs substrates for protein synthesis at the ribosome. Contemporary aaRSs are partitioned in two classes called class I and class II (3). Enzymes of each class have evolved from two unrelated ancestral proteins that arose previous to the last universal common ancestor (LUCA) and are thought to have had a broad specificity for tRNAs and amino acids (4,5). Generation of the current aaRSs was proposed to have occurred by multiple successive events of gene duplication and diversification, paralleled by a progressive narrowing of specificity for tRNAs and amino acids by the newly arising enzymes (4,6). Whereas these events are ancient, predating the apparition of the LUCA, other more recent events have sprinkled genomes of the three domains of life with duplicated aaRSs genes of which only a few have been empirically characterized (7–9). These duplicated aaRSs were observed to have diverged evolving distinct features. In some other cases, divergence has originated truncated aaRS paralogs that do not conserve the original aminoacylation function and have adopted new roles (10–12).

AaRSs are modular proteins. The catalytic domain of class I and class II enzymes catalyzes the aminoacylation reaction in two steps: the activation of the amino acid by ATP and the subsequent transfer of the amino acid moiety to the acceptor end of the tRNA (13). During the evolutive diversification of aaRSs other domains have been appended to this catalytic module. Some of the appended domains play accessory roles assisting the canonical aminoacylation reaction (i.e. by interacting with tRNA), whereas others perform a variety of functions in many cases not related to translation (14). Some aaRSs contain editing domains appended to the catalytic domain that provide a proofreading step to the aminoacylation reaction, thus contributing to the correct pairing of tRNAs with their cognate amino acid and to the overall fidelity of translation. The necessity for proofreading comes from the insufficient discrimination capacity of the active site of these aaRSs which, with a certain rate activates near-cognate amino acids and misacylates cognate tRNAs with them (15). Misacylated tRNAs are thus carriers of non-cognate amino acids and need to be hydrolyzed (edited) to prevent mistranslation (i.e. the misincorporation of amino acids to nascent polypeptides at the ribosome), which in general provoke detrimental effects (15). Crucial to translational fidelity, proofreading either occurs after the first step of the aminoacylation reaction (pre-transfer editing) or once the amino acid is bound to the acceptor end of the tRNA (post-transfer editing). The latter typically occurs at specific editing domains and requires the translocation of the acceptor end of the misacylated tRNA from the synthetic active site in the catalytic domain to a hydrolytic editing site located 30–40 Å away (16,17). Released aminoacyl-tRNAs may also be edited *in trans* most commonly by stand-alone proteins often homologous to editing domains of aaRSs (18–21).

Threonyl-tRNA synthetase (ThrRS) is a dimeric class II aaRS with proofreading activity. Specific recognition of the amino acid substrate at the catalytic site of ThrRS is based on the interaction of the hydroxyl group in the side chain of threonine with a zinc atom universally present in the active site of these enzymes (22,23). This permits the discrimination of isosteric valine, with a methyl group in the side chain that does not interact with zinc, but not of serine which, similar to threonine, contains a hydroxyl group in the side chain (22). Thus, with a certain frequency, ThrRS activates Ser and charges tRNA^Thr^ with Ser. Bacterial and eukaryotic cytoplasmic ThrRSs contain an N-terminal editing domain that hydrolyzes Ser-tRNA^Thr^ (16,24), whereas in some archaea, editing of this misacylated intermediate is mediated by trans-acting factors (19,25).

We observed that in organisms of the three domains of life, genes encoding aaRSs are very frequently duplicated and in some cases triplicated. In this work we have investigated how duplicated ThrRSs have evolved post-duplication, focusing on the asymmetric acquisition or degeneration of functional abilities, their correlation with some sequence features and how the function is partitioned between these divergent enzymes. Using the model cyanobacterium *Anabaena* we show that duplicated ThrRSs, named T1 and T2, have evolved distinct expression profiles and functional features, some of which correlate to specific sequence insertions/deletions. Evidence demonstrate that T1 is constitutively expressed, it is equipped with aminoacylation and editing activities and that T1 homodimers are responsible for Thr-tRNA^Thr^ production in zinc sufficiency, yet in low zinc T1 dimers dissociate into monomers with no aminoacylation activity. T2 expression is induced by low zinc and it is active for aminoacylation in these conditions though it is editing-defective and produces mischarged Ser-tRNA^Thr^ at a high rate. We present *in vivo* and *in vitro* evidence showing that T2 forms homodimers and also heterodimers with subunits of *apo*-T1 that provide editing activity *in trans*. Thus, three dimeric ThrRS isoforms, (T1)_2_, (T2)_2_ and *apo*-T1-T2, with distinct functional properties are assembled according to metabolic conditions. We propose that this mechanism for generating oligomeric aaRSs variants that best fit with the cell status is extensive to other organisms with duplicated oligomeric aaRSs.

## MATERIALS AND METHODS

### Organisms and growth conditions

**Figure 1.**
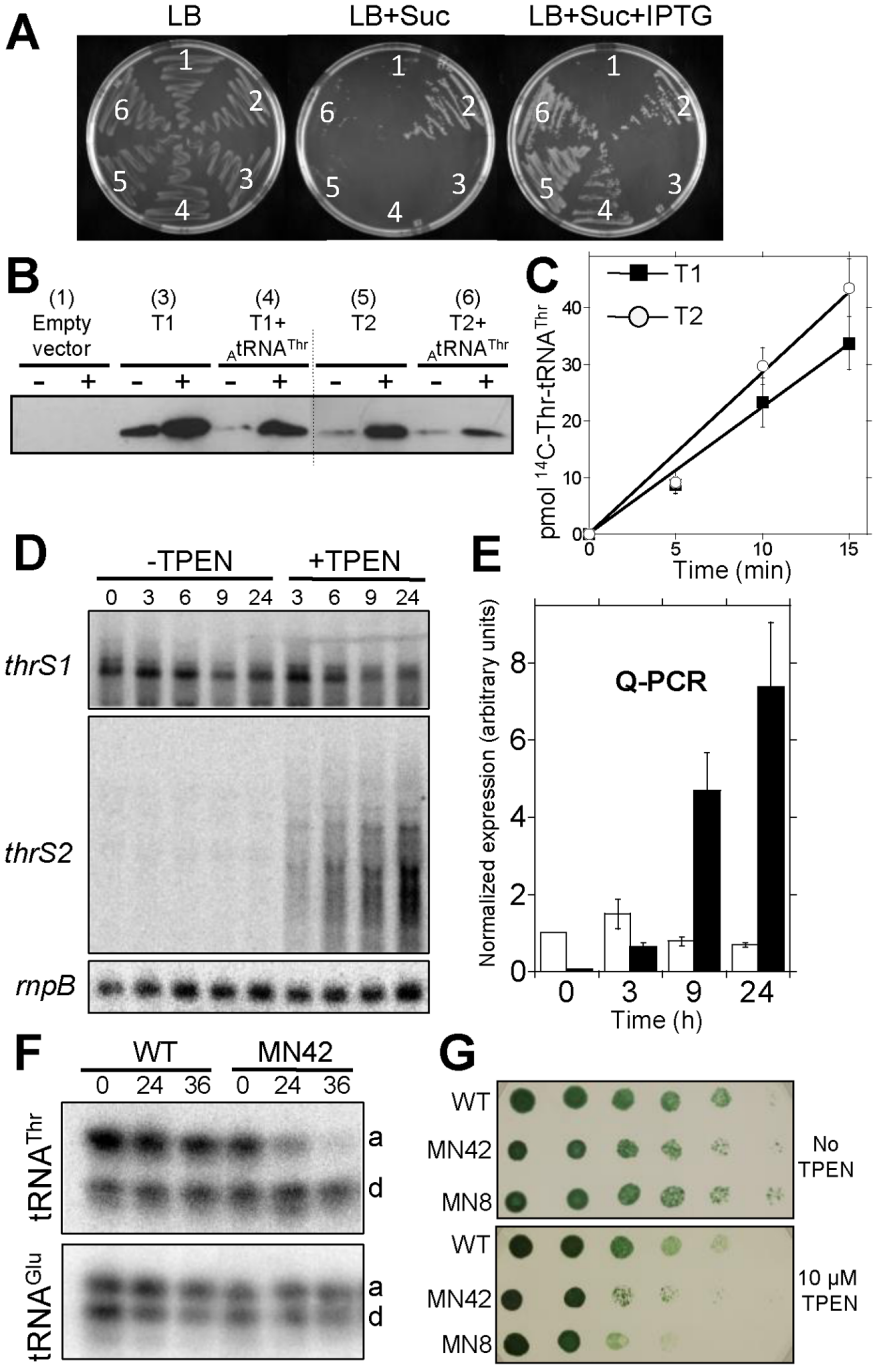
Functional characterization of T1 and T2. (**A**) Complementation assay of the *E. coli* conditional mutant IBPC6881(pSGUB4). Derived strains containing the empty vector pTrc99A (sector 1) or derived plasmids expressing the *E. coli* ThrRS (sector 2), T1 (sector 3), T1 and *Anabaena* tRNA^Thr^(CGU) (sector 4), T2 (sector 5), T2 and *Anabaena* tRNA^Thr^(CGU) (sector 6) were streaked on the indicated media. (**B**) Lack of complementation is not due to a deficient expression of T1 in *E. coli*. Western blot of extracts were carried out using 10 μg protein extracts from the strains used in the complementation assay that express the indicated products, cultured (+) or not (−) with 1 mM IPTG and incubating with anti-hexahistidine antibodies. Numbers indicate the corresponding sector for each strain in the complementation assay. (**C**) Aminoacylation assay with recombinant T1 or T2 proteins. Assays were carried out in a volume of 22 μl and contained 50 nmol of T1 or T2 and 5 μM *Anabaena* tRNA^Thr^(CGU). Average values ± s.d. of three independent experiments are represented. (**D**) Northern blot with RNA from *Anabaena* cells subjected or not to incubation in the presence of chelating agent TPEN for the indicated period of time (in hours). Genes used as probes are indicated. (Northern assays of *thrS2* in these conditions have been published elsewhere (38), and it is only shown here for the sake of comparison). (**E**) Quantitative Real-Time PCR analysis of transcripts of *thrS1* and *thrS2* from cells incubated or not with 20 μM TPEN for the time indicated in hours in the horizontal axis. Levels of the *thrS1* gene in cells not incubated with TPEN (t = 0) were assigned the arbitrary value of 1 and all other measures were expressed in proportion to this value. Empty and solid bars correspond to *thrS1* and *thrS2*, respectively. Average values ± SE of three independent biological replicas are represented. (**F**) Total tRNA (2 μg) from WT *Anabaena* cells or the deletion mutant MN42 (*ΔthrS2*) cultured for the indicated time (in hours) in the presence of 20 μM TPEN were resolved in denaturing urea-acrylamide acid gels, transferred to nylon membranes and hybridized with the indicated probes. Positions of aminoacylated tRNA and deacylated tRNA are labeled ‘a’ and ‘d’, respectively. (**G**) Serial dilutions of cell suspensions of WT *Anabaena*, the deletion mutant MN42 (*ΔthrS2*) or the insertion mutant MN8 (*thrS2::C.S3*) were spotted onto BG-11 solid medium supplemented or not with 10 μM TPEN as indicated. All experiments were repeated at least three times with identical results.

*Anabaena* sp. PCC 7120 (also known as *Nostoc* sp. PCC 7120) was routinely grown at 30°C and 75 μmol photon m^−2^ s^−1^ in BG-11 medium (26). When needed antibiotics were supplemented at the following concentrations: 5 μg ml^−1^ streptomycin, 5 μg ml^−1^ spectinomycin, 25–50 μg ml^−1^ neomycin. When indicated, TPEN (*N,N,N’,N’*-tetrakis(2-pyridilmethyl)ethylenediamine) was added to liquid cultures at a final concentration of 20 μM. The complementation assay described in Figure 1A was carried out using the *E. coli* conditional mutant IBPC6881(pSGUB4), whose chromosomal *thrS* gene is disrupted and relies on a *thrS* gene resident in plasmid pSGUB4 for survival (27). pSGUB4 carries a *sacB* gene, which is lethal in the presence of sucrose. Plasmids used for complementation (pCMN15, pCMN16, pCMN17, pCMN18 and pCMN19) are derivatives of pTrc99A. Plasmids and oligonucleotides used in this work are listed in Supplementary Tables S1 and S2, respectively.

*E. coli* DH5α was routinely used for cloning purposes and BL21(DE3) for overexpression of proteins.

### Acid urea polyacrylamide gel electrophoresis

tRNA was purified in acidic conditions from 40 ml cultures of *Anabaena*. Cell pellets were resuspended in TL buffer (0.3 M sodium acetate, 10 mM EDTA pH 4.3), incubated with TRIsure (Bioline) and chloroform, nucleic acids in the aqueous phase were recovered by precipitation with 65% isopropanol and resuspended in 10 mM sodium acetate pH 4.5, 1mM EDTA. RNA was loaded on 8 M urea, 6.5% acrylamide gels, electrophoresed as described (28), transferred to Genescreen Plus membranes (Dupont) and hybridized to (^32^P)-labeled probes.

### Purification of recombinant proteins by affinity chromatography and Western blot

Genes *thrS1* and *thrS2* from *Anabaena* were cloned in the pCMN28b vector (29) in frame with the sequence encoding a Strep-TagII and introduced in *E. coli* BL21(DE3). For overexpression, cells were cultured at 37°C to an OD (600 nm) of 0.5–0.7, supplemented with 1 mM IPTG and incubated for 3–5 h at 37°C. After harvesting by centrifugation cells were resuspended in 100 mM Tris-HCl pH 8, 150 mM NaCl, 10% glycerol and 1 mM phenylmethanesulfonyl fluoride (PMSF), extracts were prepared by lysis using a French Press and subjected to purification in StrepTrap HP columns (GE Healthcare) following the instructions of the manufacturer.

For purification of His-tagged proteins by Ni-NTA chromatography, cell extracts were prepared in 100 mM Tris-HCl pH 8, 150 mM NaCl, 10% glycerol or 20 mM sodium phosphate pH 7.4, 500 mM NaCl and applied to His-Trap columns (GE Healthcare). Chromatography was performed following the indications of the manufacturer.

Western blots were performed by resolving proteins by SDS-PAGE and transferring them to Hybond-P PVDF membranes (GE Healthcare) that were incubated with anti-His antibodies (Qiagen) or anti-StrepTag-II antibodies (IBA).

### tRNA preparation and aminoacylation assays

Gene encoding *Anabaena* tRNA^UGU(1)^ was modified to introduce a CCA sequence in the 3′-end upon cloning in pCMN28b and the resulting plasmid introduced in *E. coli* BL21(DE3). For overexpression, cells were cultured in LB supplemented with 2% glucose at 30°C to an OD (600 nm) of 0.5, washed and re-inoculated in LB with no glucose, supplemented with 1 mM IPTG and further incubated at 30°C for 16 h. After harvesting by centrifugation, cells were resuspended in TL buffer (0.3 M sodium acetate pH 4.3, 10 mM EDTA), extracted twice with phenol pH 4.5 (Amresco) and nucleic acids were recovered by precipitation with 2 volumes of absolute ethanol. One sample was resuspended in 200 mM Tris-acetate pH 8.5 and incubated 1 h at 37°C to be used as a control of deacylated tRNA. The rest of the samples was resuspended in DEPC–H_2_O and subjected to sequential precipitation with isopropanol. First, high molecular weight nucleic acids were eliminated by precipitation with 30% isopropanol and total tRNA was subsequently recovered by precipitation with 60% isopropanol. Pellets were resuspended in RNase-free water, heated at 70°C for 3 min and cooled slowly for refolding. The concentration of *Anabaena* tRNA^Thr^ in our preparations was determined by plateau aminoacylation assays (see below). Aminoacylation assays were carried out as described (30). Briefly, 22 μl reactions containing 100 mM HEPES pH 7.2, 20 mM KCl, 30 mM MgCl_2_, 5 mM ATP, 0.1 mg ml^−1^ BSA, 0.5 mM di-thiotreitol (DTT), 5 μM tRNA, 50–250 nM enzyme and 120 μM (^14^C)-L-Ser or 20 μM (^14^C)-L-Thr were incubated at 30°C and stopped on filters soaked with 5% trichloroacetic acid. For plateau aminoacylation assays the enzyme concentration used was 400 nM.

### Deacylation assays

Ser-tRNA^Thr^ was produced in plateau aminoacylation assays using editing-defective T2ΔN enzyme. 22 μl assays contained 10 μM tRNA, 5 μM enzyme, 20 μM ^3^H-L-Ser and 4 U ml^−1^ yeast inorganic pyrophosphatase. Reactions were incubated for 2 h at 30°C and stopped upon addition of 2% acetic acid. tRNA was extracted twice with phenol pH 4.5 and precipitated with 2 volumes of ethanol and incubation for 12 h at −80°C. Pellets were resuspended in 50 mM phosphate buffer pH 5.0 and Ser-tRNA^Thr^ was quantitated using a scintillation counter. Deacylation reactions contained 150 nM Ser-tRNA^Thr^, 50 nM enzyme, 150 mM KHPO_4_ pH 7. 5, MgCl_2_, 0.1 mg ml^−1^ BSA, 1 mM DTT and 4 U ml^−1^ inorganic pyrophosphatase.

### Gel filtration chromatography and metal substitution

Cromatographies were performed using 100 mM Tris pH 7,5, 150 mM NaCl, 5 mM DTT as chromatography buffer supplemented as indicated in each experiment in a Superdex 200 10/300 GL column (GE Healthcare). Aliquots of 0.5 ml were collected and assayed for tRNA^Thr^ aminoacylation. For metal substitution assays, preparations of T2 containing 25 μM protein were incubated with 5 mM EDTA for 12 h at 4°C. 100 μl were applied to a Superdex 200 column equilibrated with Chelex 100-treated chromatography buffer containing the indicated metal salt at 10 μM final concentration. Aminoacylation activity was determined in fractions corresponding to the peak that were subsequently subjected to buffer exchange in PD-10 columns equilibrated with 10 mM HEPES pH 7.5, 50 mM NaCl previously depleted of metals by filtration through Chelex-100 resin twice. Metal content in the eluted fractions was determined by ICP-MS at the CITIUS facility (Universidad de Sevilla).

### Zinc chelator competition assays

To produce the *apo*-form of Strep Tag-II tagged T1 or T2 proteins purified in streptavidine columns as described above were further purified by HiTrap Heparin HP columns (GE Healthcare) using 100 mM Tris-HCl pH 7.8, 50 mM NaCl, 5 mM DTT and 5 mM EDTA as a chromatography buffer. Protein charged columns were transferred into an anaerobic chamber and fractions were eluted using a buffer containing 10 mM Hepes pH 7 and 500 mM NaCl. Protein content was measured by measuring the absorbance at 280 nM and a 5,5′-dithio-*bis*-(2-nitrobenzoic acid) (DTNB) assay was performed to confirm the reduced state of the protein. Zn(II) was added incrementally to a mix containing the protein and Mag-Fura-2 and the decrease in the absorbance at 366 nm was monitored subsequent to each Zn(II) addition.

Proteins were supplemented with two equivalents of Zn(II) and 20 equivalents of NTA or HEDTA as indicated and incubated at 4°C. After 24 h, proteins were resolved on a Sephadex G-25 matrix previously equilibrated with metal-depleted buffer (all performed under anaerobic conditions). Fractions were analyzed by ICP–MS and the protein content was measured using a Bradford assay. The pH dependent zinc binding constants of NTA and HEDTA were calculated using Schwarzenbach's α-coefficient method (Equations 1 and 2) (31).

(1) \begin{equation*} K^{\prime} _{\rm A} = K_{\rm A} \alpha _{{\rm H} - {\rm L}} \end{equation*}

(2) \begin{equation*} \alpha _{{\rm H} - {\rm L}} = (1 + \beta _{{\rm H},1} [{\rm H}] + \beta _{{\rm H},2} [{\rm H}]^2 + \ldots + \beta _{{\rm H},{\rm n}} [{\rm H}]^{\rm n} )^{ - 1} \end{equation*}

where *K*_A_′ is the pH dependent zinc affinity constant, *K*_A_ the absolute zinc affinity constant, α_H–L_ is Schwarzenbach's α-coefficient, *β*_H,1_ = 10^p*K*a1^, *β*_H,2_ = 10^p*K*a1 + p*K*a2^ etc., [H] = 10^−pH^. NTA has an absolute affinity constant for zinc of 10^10.66^ M^−1^ and sequential acid dissociation constants p*K*a1 = 9.73, p*K*a2 = 2.49, p*K*a3 = 1.89 (31). HEDTA has an absolute affinity constant for zinc of 10^14.6^ M^−1^ and sequential acid dissociation constants p*K*a1 = 9.87, p*K*a2 = 5.38, p*K*a3 = 2.62 (31).

### Amino acid analysis

Amino acid determination was performed after derivatization as described in (32). Briefly, pellets from 50 ml cultures were collected by centrifugation, resuspended and incubated for 1 h in 1 ml 0.1 N HCl on ice and further centrifuged at 12,000 x *g* for 10 min at 4°C. A volume of 60 μl of the supernatant was derivatized by mixing with an equal volume of ethanol:H_2_O:triethanolamine:phenylisothiocyanate 7:1:1:1, incubated at room temperature for 30 min, and dried under flowing N_2_. The pellet was resuspended in 60 μl of 4 mM sodium phosphate (pH 7.4), 2% (vol/vol) acetonitrile and resolved by reverse phase HPLC using a LiChrospher 100 RP-18 (4 mm x 125 mm) column (Merck). Chromatography was performed at 1.5 ml min^−1^, 46°C in an Elite LaChrom (Hitachi) system by a step-wise gradient of solvent A, containing 70 mM sodium phosphate (pH 6.55) and 2% acetonitrile (vol/vol) and solvent B, containing 50% (vol/vol) acetonitrile. AA-S-18 (Sigma) was used as internal standard. A cytoplasmic volume of 125 μl per mg chlorophyll (33) was considered for calculations of intracellular concentration of amino acids.

### Bioinformatics and phylogenetic methods

Genomes with redundant aaRS genes were extracted by interrogating the JGI database (https://img.jgi.doe.gov/) as of 06.05.2015.

For the phylogeny of ThrRSs, all 9629 ThrRS sequences available at Refseq-NCBI (as of October, 2014) were retrieved using a copy of *Anabaena* (*Nostoc*) sp. PCC 7120 (gi 637230694) as query for a DELTA-BLAST search (34). Sequences were MAFFT aligned (35) and Jalview (36) was used to remove incomplete sequences and to reduce redundancy (99% threshold). Multiple alignment was then trimmed under stringent conditions to remove saturated positions, gaps and constant sites resulting in 2654 sequences and 151 variable positions (34). Phylogenetic tree was reconstructed using RAxML (37).

## RESULTS

### Duplicated genes encoding aaRSs are common in the three domains of life

26,837 genomes from organisms of the three domains of life were surveyed for duplicated or triplicated genes encoding aaRSs and ca. 25,913 cases were observed (Data set S1). Genes encoding ThrRS were found duplicated or triplicated in more than 1,000 genomes, of which about 900 were bacteria including one third of cyanobacterial species and a variety of Gram-negative and Gram-positive bacteria. Such duplications were the result of multiple evolutionary events that, in many cases, preceded the diversification of phyla (Supplementary Figure S1). To investigate the role of duplicated *thrS* genes, the cyanobacterium *Anabaena* sp. PCC 7120 (hereafter *Anabaena*) was chosen as a model. *Anabaena* genes *thrS1* and *thrS2* encoding enzymes T1 and T2, respectively, were both shown to be functional by complementation of an *E. coli* conditional mutant (27) (Figure 1A), and by *in vitro* aminoacylation assays of tRNA^Thr^ (Figure 1C). Interestingly, T1 only complemented the *E. coli* mutant when co-expressed with a tRNA^Thr^ from *Anabaena* (Figure 1A, compare sectors 3 and 4 and Figure 1B), evidencing a distinct ability of the two enzymes to charge tRNA^Thr^ from distinct sources and pointing to the existence of critical structural differences between both proteins. Functional redundancy of *thrS1* and *thrS2* was investigated by construction and analysis of *Anabaena* mutants. Knock-out mutants of *thrS2* gene were readily obtained and have been reported (29,38). By contrast, no *thrS1* mutants could be obtained despite repeated efforts, implying that *thrS1* is essential. Repeated attempts to replace *thrS1* by *thrS2* were also unsuccessful, indicating that these genes are not exchangeable and not functionally redundant.

### Differential expression of *thrS* genes in *Anabaena*

Gene *thrS2* forms an operon with four flanking genes, which are repressed under standard growth conditions and induced by zinc limitation. This response is controlled by Zur, a zinc-sensing regulator of the FUR family that represses two promoters of the operon, one just upstream of *thrS2* (29,38). Here, the expression of *thrS1* was analyzed in cells cultured under standard growth conditions or subjected to zinc limitation (29,38). Unlike *thrS2*, *thrS1* showed a moderate decay of expression as zinc limitation advanced, to reach a level of about half the initial one (Figure 1D,E). Quantitative assessment of the expression of both genes by real-time Q-PCR (Figure 1E) showed that under standard growth conditions *thrS2* expression was almost undetectable, and *thrS1* transcript was about 20-fold more abundant. By contrast, 24 h after the onset of zinc deficiency, *thrS2* transcript abundance increased 200-fold to become 10-fold more abundant than the *thrS1* transcript.

Differential expression profiles suggested distinct tasks for T1 and T2, prompting us to investigate the role of each enzyme in *Anabaena* cells during the course of zinc deficiency. The proportion of charged versus uncharged tRNA^Thr^ was assessed in time-course experiments using acid urea polyacrylamide gel electrophoresis. Whereas in WT-*Anabaena* the level of aminoacylated tRNA^Thr^ only showed a slight decay as zinc deficiency advanced, in a *thrS2* deletion mutant, this level decreased 5-fold (Figure 1F). By contrast, the proportion of charged tRNA^Glu^ showed little variation for both strains. This suggested a major role for the T2 enzyme in the aminoacylation of tRNA^Thr^ under zinc limitation. In line with this, growth of insertion or deletion mutants of *thrS2* was impaired after prolonged culture (10–15 days) on solid medium supplemented with TPEN, a divalent cation chelator (Figure 1G). These results were consistent with the expression pattern observed for *thrS1* and *thrS2* genes, and evidence a prominent role for the T2 enzyme in the aminoacylation of tRNA^Thr^ under low zinc.

### T1 and T2 use zinc as cofactor

**Figure 2.**
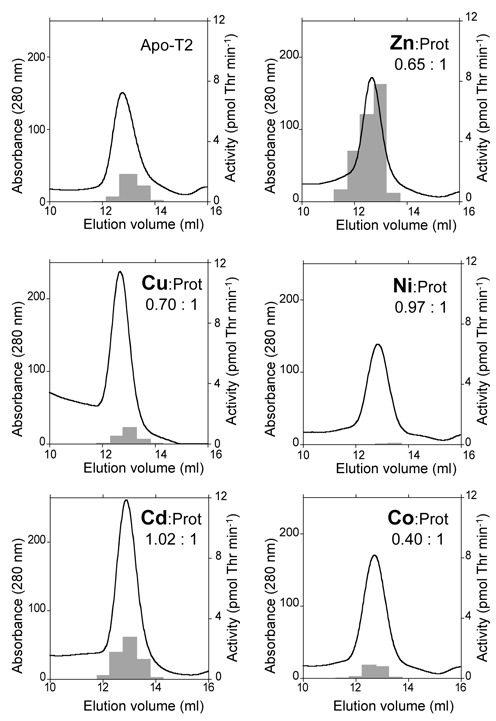
*In vitro* replacement of the metal cofactor of T2. Pure preparations of recombinant T2 protein at concentration 25 μM were incubated with 5 mM EDTA and subsequently subjected to gel filtration in a Sephadex S-200 column equilibrated with Chelex 100-treated buffer containing 150 mM NaCl, 100 mM Tris-HCl pH 7.5, 1 mM DTT, supplemented with the metal indicated in each panel at a concentration of 5 μM. Aminoacylation activity was determined in fractions that contained protein and it is represented as gray bars. Fractions corresponding to the peak were subjected to buffer exchange to eliminate metals from the buffer and analyzed by ICP–MS. Numbers indicate the metal:protein ratio of the peak fractions.

All ThrRSs characterized to date are reported to be metalloenzymes with a zinc cofactor in the catalytic site essential for activity (23,39–41). Thus, the observation that T2 was predominantly active *in vivo* under low zinc was unexpected and pointed at the existence of mechanisms to maintain its activity despite the low concentration of zinc. Sequence comparisons showed that residues identified as zinc-ligands in other ThrRSs (23,39,40) are conserved in T1 and T2 (Supplementary Figure S2), and metal analyses by inductively coupled plasma mass spectrometry (ICP–MS) revealed that heterologously expressed T1 and T2 contained approximately two atoms of zinc per dimer, rising to approximately three atoms per dimer for T2 following incubation with zinc (see Supplementary Figure S3). Based on this, the hypothesis that T2 could bind zinc with higher affinity than T1 was tested. However, competition experiments with chelating agents did not reveal large differences in the affinity of T1 and T2 for zinc, which was in the range of 10^−10^– 10^−12^ M for both proteins (Supplementary Figure S3B–D). A well-documented alternative strategy for acclimation to low-zinc environments consists of replacing zinc metalloproteins by isoforms that use an alternative metal cofactor (42). This prompted us to check whether T2 would be active using another metal. To test this, T2 was prepared in the *apo* form by prolonged incubation with EDTA and it was re-metallated with a variety of divalent metals by buffer exchange in gel filtration. As shown in Figure 2, these *in vitro* treatments allowed metallation of T2 with Zn, Ni, Cu or Cd in proportions close to one atom per monomer, or somewhat less efficiently with Co. However, except for the zinc-containing enzyme, all other metal-substituted preparations showed a tRNA charging activity similar to the residual activity of the *apo*-protein (Figure 2) indicating that T2 is not functional with an alternative metal cofactor.

### T1 dissociates upon cofactor loss

**Figure 3.**
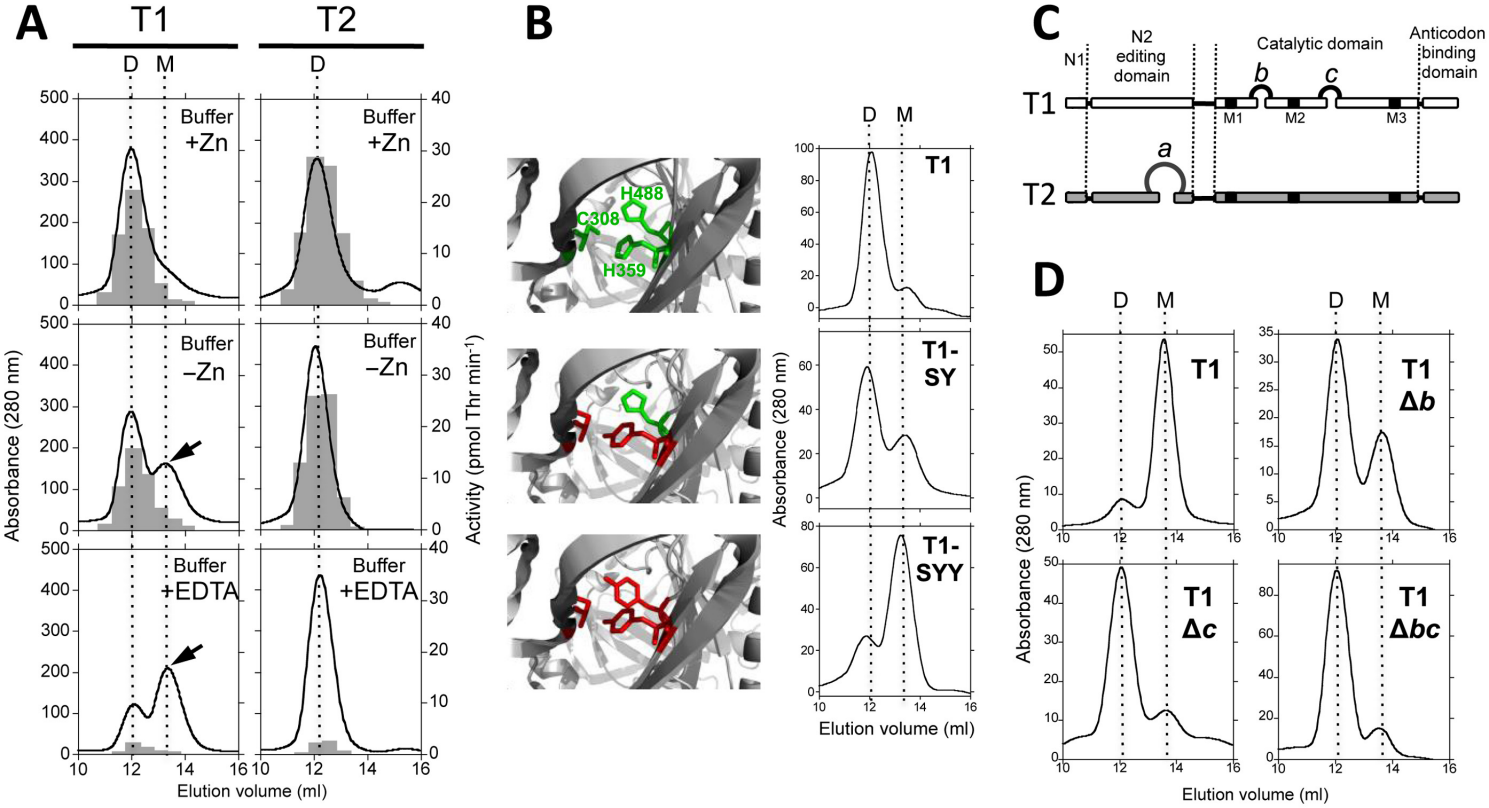
Cofactor loss promotes dissociation of T1. (**A**) Gel filtration assays of recombinant T1 and T2 proteins in buffer containing 150 mM NaCl, 100 mM Tris-HCl pH 7.5, 1 mM DTT, supplemented with 5 μM ZnSO_4_ (top panels) or 5 mM EDTA (bottom panels). ‘D’ and ‘M’ indicate the elution volume corresponding to the size of a dimer (150 kDa) or a monomer (75 kDa), respectively (**B**) Gel filtration assays in buffer supplemented with 5 μM ZnSO_4_ of WT, double mutant T1-SY (Cys308Ser and His359Tyr) and triple mutant T1-SYY (containing the additional mutation His488Tyr). Pictures on the left are based on the *E. coli* ThrRS structure (PDB code 1QF6) and represent the zinc-binding pocket showing in green the zinc coordination residues and in red residues mutated in the SY and SYY proteins. (**C**) Diagram showing the position of insertions *b* and *c* of T1 and insertion *a* of T2. "M1–3" indicate the conserved motifs of the catalytic domain of class II aaRSs. (**D**) Gel filtration of wild-type T1 or mutant proteins containing the indicated mutations using chromatography buffer supplemented with 5 mM EDTA.

To better understand the traits that make enzymes T1 and T2 best suited for functioning under zinc sufficiency and deficiency, respectively, their physico-chemical properties were analyzed. The quaternary states of T1 and T2 were analyzed by gel filtration chromatography (Figure 3A). When 5 μM ZnSO_4_ was present in the chromatography buffer, both proteins eluted with an estimated molecular weight of ca. 150 kDa, consistent with the expected MW of dimeric ThrRSs (Figure 3A). Omission of zinc or addition of EDTA to the buffer in chromatographies of the T1 protein generated a novel peak, devoid of aminoacylation activity, corresponding to the size of a monomer (Figure 3A). By contrast, T2 eluted as a dimer in all conditions. These results indicated that T1 dissociated upon removal of the zinc cofactor, which is in sharp contrast to the stability of T2 dimers. To confirm this, T1-derived mutant proteins carrying substitutions in the coordination residues for zinc were generated and tested. By contrast to wild-type T1, which elutes as a dimer when zinc is present in the buffer, double mutant T1-SY (containing mutations Cys308Ser and His359Tyr) and triple mutant T1-SYY (containing the additional mutation His488Tyr) eluted partly or mostly as monomers in gel filtration, respectively (Figure 3B), confirming a role for zinc in the preservation of the dimeric state of T1. To check whether dissociation of T1 is reversible, *apo*-T1 was prepared by prolonged incubation with EDTA and subjected to gel filtration using a buffer supplemented with 5 μM zinc. As shown in Supplementary Figure S4A, re-metallation promoted dimerization of T1. Combined, these results indicate that the oligomeric state of T1 is governed by the allosteric occupancy of the zinc pocket.

T1 and T2 sequences were compared in search for features that could correlate to the distinct stability of their dimers. The catalytic domain of T1 contains two short insertions of 6 and 3 amino acids, named *b* and *c*, which are absent in T2 (Figure 3C and Supplementary Figure S2). Individual or simultaneous deletion of *b* or *c* generated mutant proteins T1Δ*b*, T1Δ*c* and T1Δ*bc*, which unlike wild-type T1, eluted mostly as dimers in gel filtration in the presence of EDTA (Figure 3D). These T1-derived mutant proteins were in this respect similar to T2. T1Δ*b*, T1Δ*c* and T1Δ*bc* were active, showing respectively 120 ± 18, 60 ± 9 and 37 ± 6% (average ± s.d.) of the aminoacylation activity of wild-type T1. These results provide a structural basis for the dissociation of T1 and indicate that insertions *b* and *c* are somehow involved in the dynamic events linking cofactor loss to the dissociation of T1.

### T2 is impaired in editing activity

**Figure 4.**
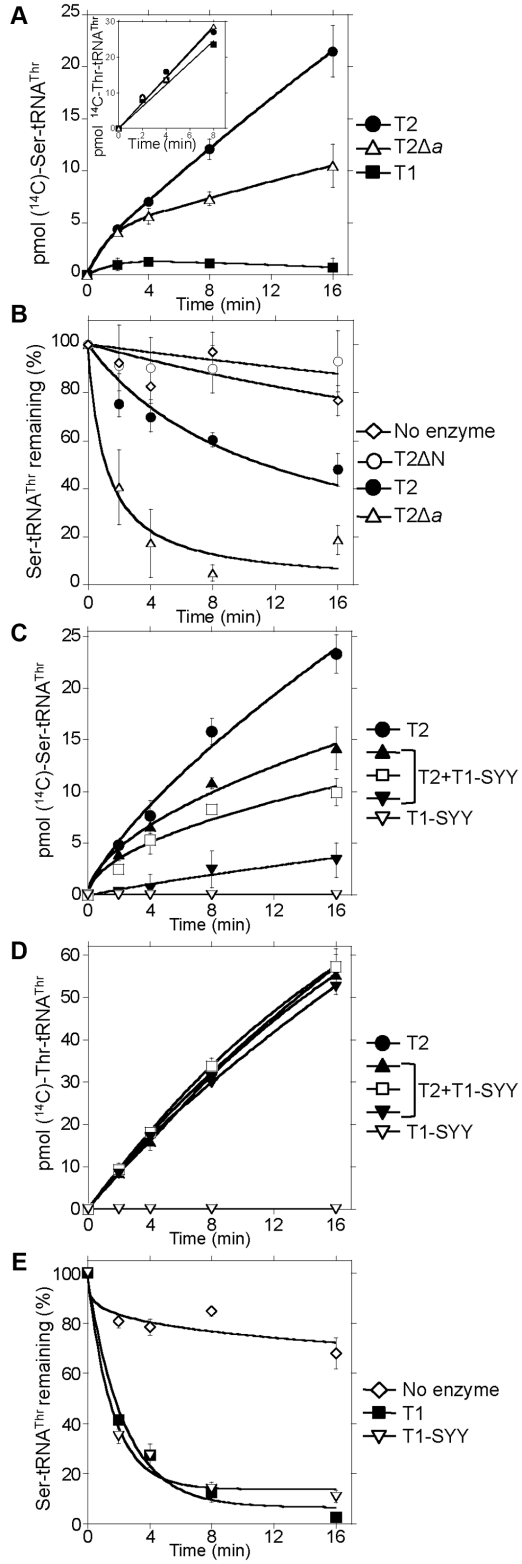
T2 misacylates tRNA^Thr^ with L-Ser. (**A**) Aminoacylation assays were carried out in a volume of 22 μl containing 5 μM *Anabaena* tRNA^Thr^ and 120 μM (^14^C)-radiolabeled L-Ser and 200 nM of enzymes T1 (solid squares), T2 (solid circles) or T2*Δa* (triangles). A similar assay using 20 μM (^14^C)-radiolabeled L-Thr is represented in the inset. (**B**) 150 nM purified Ser-tRNA^Thr^ was incubated in deacylation assays with 50 nM T2 (solid circles), T2*Δ*N (empty circles), T2Δ*a* (triangles) or no enzyme (diamonds). (**C**) Aminoacylation assays of tRNA^Thr^ with L-Ser were performed like in (**A**) and contained 200 nM T2 (monomer concentration, solid circles), 200 nM T1-SYY (empty inverted triangles) or mixtures of T2 (200 nM monomer) and T1-SYY in proportions 1:1 (solid triangles), 1:2 (empty squares) or 1:5 (solid inverted triangles). (**D**) Aminoacylation assays of *Anabaena* tRNA^Thr^ with 20 μM (^14^C)-radiolabeled L-Thr and 20 nM T2 (monomer concentration, solid circles), 20 nM T1-SYY (empty inverted triangles) or mixtures of T2 (20 nM monomer) and T1-SYY in proportions 1:1 (solid triangles), 1:2 (empty squares) or 1:5 (solid inverted triangles) were carried out as in (**A**). (**E**) Purified Ser-tRNA^Thr^ was incubated in deacylation assays with 100 nM T1 (monomer concentration, solid squares), 100 nM T1-SYY (inverted empty triangles) or no enzyme (diamonds). Average values ± s.d. of three independent experiments are represented.

Given the stability of T2, its inability to replace T1 was intriguing. T2 contains an extra 20 amino acid sequence (insert *a*, residues 128–147) in the N2 editing domain, close to residues C154 and H158 (C182 and H186, respectively in the *E. coli* enzyme), important for editing (Figure 3C and Supplementary Figure S2) (16). Potential effects of this insertion on editing were tested by monitoring the production of tRNA^Thr^ aminoacylated with near-cognate L-Ser. As shown in Figure 4A, T2 produced Ser-tRNA^Thr^ at a measurably higher rate than T1 suggesting that T2 is impaired in post-transfer editing activity and pointing to insert *a* as the possible cause. To test this, we deleted insert *a* from T2. The mutant protein T2Δ*a* showed intact aminoacylation activity as it charged tRNA^Thr^ with Thr at a similar rate than T2 (see inset in Figure 4A) but produced misacylated Ser-tRNA^Thr^ at a lower rate (Figure 4A), indicating an improved editing activity for T2Δ*a*. Further support for this was obtained by direct assessment of editing activity measuring deacylation of purified Ser-tRNA^Thr^ (Figure 4B). These results implied that the evolutive acquisition of insert *a* has impaired the ancestral editing activity of T2. It is worth noting that although impaired, T2 is not totally devoid of editing activity (compare the deacylation curve of T2 with that of T2ΔN mutant, which lacks subdomains N1 and N2 and has null editing activity) (Figure 4B).

Some mitochondrial enzymes like PheRS and LeuRS have been shown to compensate the evolutive loss of post-transfer editing activity by increased discrimination at their active site (43–45). To check whether T2 has evolved similarly, activation of Thr and Ser by T1 and T2 was analyzed by means of ATP-PPi exchange assays. As shown in Table 1, T2 revealed 5- and 4-fold more efficient than T1 for activation of Thr and Ser, respectively (catalytic efficiency is defined as *k_cat_/K_M_*). However the specificity factor for Thr over Ser (defined as the ratio of catalytic efficiencies for Thr and Ser) was in the same range for T1 and T2 (300 versus 400, respectively) and far from the value expected for a highly specific enzyme that should be at least 3 × 10^3^ (46). Therefore, in the case of T2, impairment of post-transfer editing was not associated to an enhancement of discrimination by the active site, as described for some mitochondrial aaRSs.

**Table 1.** Steady-state kinetic constants for ATP-[^32^P]PPi exchange by T1 and T2 enzymes

	Thr			Ser			
	*K*_M_ (mM)	*k*_cat_ (s^−1^)	*k*_cat_/*K*_M_ (mM^−1^ s^−1^)	*K*_M_ (mM)	*k*_cat_ (s^−1^)	*k*_cat_/*K*_M_ (mM^−1^ s^−1^)	Specificity factor (*k*_cat_/*K*_M_)Thr/(*k*_cat_/*K*_M_)Ser
T1	2.6 ± 0.3	10.0 ± 1.5	3.8 ± 0.7	274 ± 4	3.290 ± 0.014	0.0120 ± 0.0002	314
T2	0.22 ± 0.03	4.42 ± 0.13	20.1 ± 2.6	77 ± 14	3.6 ± 0.4	0.046 ± 0.010	436

Numbers indicate average ± standard deviation (n = 3).

*In vivo*, accurate discrimination of cognate versus near-cognate amino acid is also influenced by their relative abundance in the cytoplasm. For instance, discrimination by T2 could be favored if under zinc deficiency the concentration of Ser was several-fold lower than that of cognate Thr. To test this the concentrations of Thr and Ser in the cytoplasm of *Anabaena* cells were determined and the specificity factor (defined as selectivity factor x [Thr]/[Ser]) was calculated for T1 and T2 (using for the latter the values measured in cells treated with TPEN). As shown in Table 2, the selectivity factor was very similar for T1 and T2.

**Table 2.** Concentrations of threonine and serine in cells subjected or not to zinc deficiency

	[Thr] (mM)	[Ser] (mM)	[Thr]/[Ser]		Selectivity factor (Specificity Factor x[Thr]/[Ser])
**-TPEN**	1.19 ± 0.03	0.810 ± 0.024	1.56 ± 0.06	**T1**	490
**+TPEN**	4.1 ± 0.3	3.50 ± 0.09	1.17 ± 0.08	**T2**	511

Numbers indicate average ± standard deviation (n = 3).

### Trans-editing of Ser-tRNA^Thr^ by T1 subunits

The inability to replace T1 by T2 through genetic manipulation was consistent with the observed impairment of T2 editing activity and the low discrimination capacity of its active site. However, mutants expressing T2 at relatively high levels were easily obtained suggesting the existence of proofreading mechanisms. It was then considered whether T1 subunits could act as a *trans*-editing factor of Ser-tRNA^Thr^ produced by T2. This may also happen in the wild type in zinc deficiency as T1 expression is not null (Figure 1D,E) and T2 dimers would be expected to co-exist with some T1 subunits. For experimental approaches directed to ascertain this role for T1, we reasoned that mutant protein T1–SYY was best suited as it does not harbor zinc in the active site and it is devoid of synthetic activity, best resembling T1 subunits in low zinc (Figure 3B). As shown in Figure 4C, the production of Ser-tRNA^Thr^ by T2 decreased when T1–SYY was included in the assay, showing an inverse correlation with the concentration of T1–SYY. By contrast, the presence of T1–SYY had no effect on the aminoacylation of tRNA^Thr^ with cognate Thr catalyzed by T2 (Figure 4D). In deacylation assays of Ser-tRNA^Thr^, T1–SYY showed intact editing activity similar to that of wild-type T1 (Figure 4E). These results indicated that *in vivo* T1 subunits could act under zinc deficiency as a *trans*-editing factor of misacylated tRNAs produced by T2.

**Figure 5.**
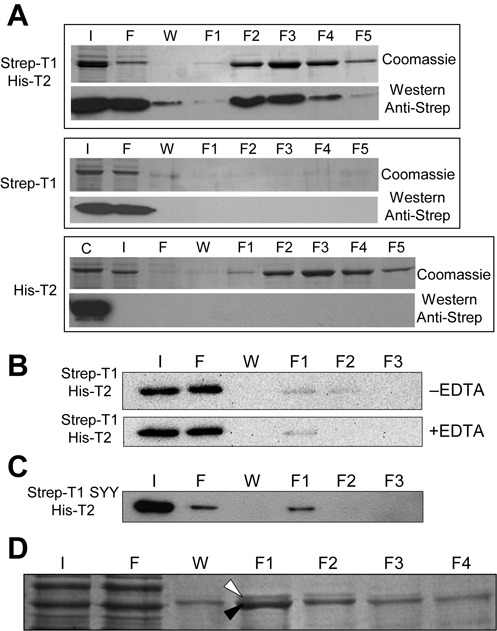
T1 and T2 form heterodimers. (**A**) Extracts form *E. coli* expressing the proteins indicated at the left were subjected to Ni-NTA affinity chromatography, and fractions were resolved by SDS-PAGE and stained with coomassie or transferred to membranes and incubated with anti-StrepTag-II antibodies, as indicated at the right. Letters ‘I, F, W and F1-F5′ indicated respectively input, flow through, wash and elution fractions 1–5. Letter ‘C’ on top of the bottom panel indicate a positive control for the western. (**B**) Panels show western blots with anti StrepTag-II antibodies. Labels and details are like in (**A**) but the chromatography was performed in the presence or absence of 5 mM EDTA (as indicated on the right) and the washing step was increased to 20 column volumes. (**C**) Labels and details are like in (**A**). (**D**) Extracts were prepared from *Anabaena* cells expressing Strep-T2 and purified through streptavidine columns. White and black arrowpoints indicate respectively T1 and Strep-T2 proteins.

To get a deeper insight on the context where *trans*-editing occurs, we tested whether dissociated T1 subunits may heterodimerize with T2. A first approach demonstrated that when co-expressed in *E. coli*, distinctly tagged T1 and T2 co-purified (Figure 5A top panel and Supplementary Figure S4B), strongly indicating that they can form heterodimers. Control experiments ruled out cross-reaction of the antibodies or unspecific affinity of the purification resin (Figure 5A middle and bottom panels and Supplementary Figure S4C,D). In those experiments *E. coli* was cultured in rich medium and T1 was most probably metallated, retaining full dimerization potential. To test whether *apo*-T1, which does not form homodimers, was able to dimerize with T2, we conducted similar experiments that included an extensive incubation of the cell extracts with 5 mM EDTA, supplementation of all buffers used for purification with EDTA, and an extended chromatography washing step. In such stringent conditions T1 co-purified with T2 (Figure 5B). Furthermore, T1–SYY mutant protein, which does not form homodimers, also co-purified with T2 from *E. coli* cells expressing both proteins (Figure 5C). These experiments strongly indicated that T1 can form heterodimers in its *apo*-form, a condition in which it does not form homodimers.

To further corroborate these data we sought for *in vivo* evidence of the existence of T1-T2 heterodimers in *Anabaena*. For this, a strain expressing T2 fused to a StrepTag-II in its N-terminus was generated. Cells were incubated with TPEN for 24 h to induce expression and extracts were purified by affinity chromatography. Eluted fractions from the chromatography contained two closely migrating bands of ca. 75 KDa that were identified as T1 (upper band) and T2 (bottom band) by mass spectrometry (Figure 5D), corroborating the existence of T1-T2 heterodimers in zinc-deficiency *in vivo*.

## DISCUSSION

### A model for the concerted function of duplicated ThrRSs

**Figure 6.**
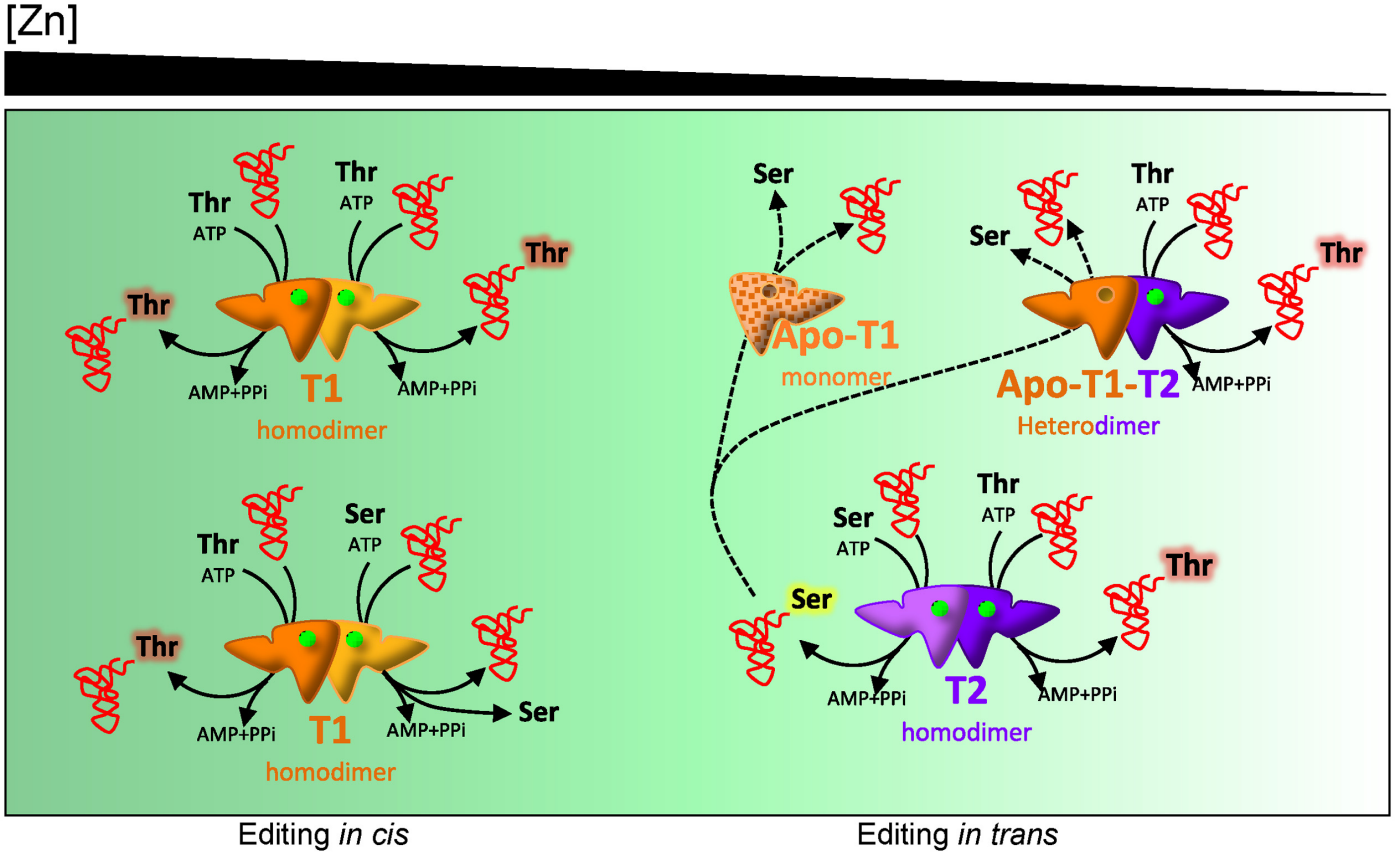
Model for the concerted action of T1 and T2. The diagram represents distinct states of the cell according to zinc availability as indicated on top of the panel. T1 is depicted in yellow-orange and T2 in purple-magenta. Green dots represent zinc atoms. Amino acids bound to the tRNA are highlighted. The *apo*-T1 monomer is colored with a dotted pattern to indicate that it may exist transiently upon dissociation of dimeric T1.

Diversification of duplicated genes is one of the leading forces in evolution and has been the subject of intense investigation and modeling (1). In this article we describe how the divergent evolution of two ThrRSs have resulted in differential expression profiles, enzymatic properties, physico-chemical features and functional roles and we identify sequence features that correlate to them. Phylogenetic analyses indicate that duplicated *thrS* genes have arisen by an early event previous to the diversification of the cyanobacterial *phylum* (Supplementary Figure S1). Billion years divergence has led to the accumulation of sequence differences, including three idiosyncratic insertions (47). Results presented in this work indicate specific non-redundant roles for duplicated ThrRSs. A model for the functioning of T1 and T2 based on our data is proposed in a diagram representing different stages of the cell according to zinc availability (Figure 6). This model proposes that under zinc sufficiency the cytoplasm would be populated only by T1 homodimers, as Zur represses T2 expression. T1 is equipped with aminoacylation and editing activities and it would produce only Thr-tRNA^Thr^. As zinc becomes scarce, release of the zinc cofactor from T1 would promote dissociation and loss of its synthetic activity. Low zinc would also determine liberation from Zur repression and expression of T2, which would be dimeric and active under these conditions. Dissociated *apo*-T1 has the capacity of associating with T2 subunits to form heterodimers. Thus, under low zinc the cytoplasm would be populated by an equilibrium of T2 dimers, *apo*-T1-T2 heterodimers and perhaps, some *apo*-T1 monomers. T2 is impaired in post-transfer editing activity, so a fraction of the aminoacyl-tRNAs released would be Ser-tRNA^Thr^. *Apo*-T1 subunits would bind and edit released Ser-tRNA^Thr^ *in trans*, preventing or limiting mistranslation under low zinc. Therefore, heterodimers formed *in vivo* under low zinc would show structural and functional asymmetry, being composed of a subunit (T2) with synthetic activity (and some residual editing activity) and a subunit (*apo*-T1) only devoted to mischarged tRNA edition.

Our results show that *in vivo*, T2 conserves aminoacylation activity under low zinc but it is not clear how (Figure 1F,G) as it does not show a comparatively high affinity for zinc (Supplementary Figure S3). This suggests the existence of unknown mechanisms for the preservation of its activity. A survey for other divalent metals that could replace zinc as cofactor indicated that only zinc supported T2 aminoacylation activity (Figure 2). However, it cannot be ruled out that alternative cofactor(s) not tested in our *in vitro* assays may replace zinc and support activity *in vivo*. Alternatively, T2 could be assisted by accessory proteins (i.e. metallochaperones) for capture and retention of zinc in the active site. Interestingly, gene *all4722*, adjacent to and co-transcribed with *thrS2*, encodes a putative metallochaperone of the COG0523 family (48). However, all attempts to demonstrate interaction between both proteins failed. The higher zinc load of T2 observed in some experiments could also be helpful for recruiting zinc to its active site when the metal is scarce (Supplementary Figure S3).

### Degeneration of the editing domain of T2

Post-transfer editing by T2 is impaired but not null (Figure 4B). The residual editing activity is consistent with the conservation of motifs HxxxH and DxCRGPH important for activity (16,24). In turn, reduced editing activity correlates with the presence of insertion *a*, a 20-amino acids long insertion in the close vicinity of motif DxCRGPH, which probably disrupts the geometry of the editing pocket. Consistent with this, the editing activity of T2 is improved by deletion of insert *a* (Figure 4A,B). From an evolutionary point of view, this constitutes an empirical resurrection of the editing function of ancestral T2. An interesting observation is that, within the cyanobacterial phylum not all organisms have conserved both ThrRSs and in species where T2 is the only enzyme, this insertion is either missing or short (6–7 amino acids) (Supplementary Figure S2). This suggests that T2 is most likely not impaired in editing in those species, suggesting in turn that a misacylating ThrRS is negatively selected as a housekeeping enzyme. Other aaRSs with degenerated post-transfer editing domains have been shown to rely on alternative proofreading mechanisms including increased discrimination at the active site (43–45), kinetic proofreading (49) or pre-transfer editing (50). Our results ruled out active site discrimination as an effective fidelity mechanism for T2 (Tables 1 and 2) but do not dismiss the operation of other mechanisms. However, these do not seem to be effective to prevent the production of Ser-tRNA^Thr^ *in vitro* (Figure 4) and also probably *in vivo*, given the repeatedly observed inability of T2 to replace T1 by genetic engineering. In the system described here, the mechanism selected to compensate the evolutive degeneration of the editing domain of T2 is post-transfer *trans*-editing by *apo*-T1 subunits.

ThrRSs sequences containing insertions at the same position as the insert *a* of T2 are also particularly frequent in *Mycoplasma* and Gram-positives of the high G+C group. Based on our data, these ThrRSs would be expected to be impaired for editing, as proposed for *Mycoplasma* ThrRS (51), implying that either these organisms have a higher tolerance for translational errors, which seem to be the case of *Mycoplasma* (49,51) or that they rely on alternative proofreading mechanisms.

### Allosteric control of T1 oligomeric state

Data presented indicate that dissociation of T1 is a reversible process controlled by zinc. Counter to other oligomeric metalloprotein complexes where a metal functions as a molecular staple by coordinating residues of distinct subunits (52), in ThrRS zinc is buried in the structure at about 15 Å off the interface, making impossible its interaction with the two subunits (22). Therefore, the regulation exerted by zinc on the oligomerization of T1 is a *bona fide* allosteric effect. Capture/release of zinc probably induces a conformational rearrangement that initiates at the zinc-binding pocket and propagates toward the interface promoting dimerization or dissociation, respectively. Our data indicate that inserts *b* and *c* are involved in the propagation of the rearrangement, so that their deletion precludes dissociation by shortcutting transmission toward the interface (Figure 3D). It must be noted that propagation of the structural change does not necessarily follow the shortest distance between the zinc-binding pocket and the dimerization interface, as insertion *c* is not located between them in the three dimensional structure (Supplementary Figure S5). Though this may appear counter-intuitive, systematic studies have demonstrated that residues far from the dimerization interface are common key players for changes in the oligomeric state of protein complexes (53). Quite interestingly, *apo*-T1 still heterodimerize with T2, implying that the T2 interface is able to accommodate the structural changes induced at the T1 interface by cofactor loss. These results provide a first insight on the structural basis for the allosteric control of the oligomeric state of T1 and its evolution. Further structural and molecular dynamics analyses could help to elucidate the sequence of events and the structural elements involved.

### Functional switch by T1

Our results indicate alternating roles for T1 *in vivo* depending on the provision of zinc. Reversible transitions in its oligomeric state promote a functional switch for T1, which oscillates from a zinc-containing homodimeric state (*holo*-T1) where it is a fully functional ThrRS equipped with synthetic and editing activities, to another state (*apo*-T1) where it is devoid of synthetic aminoacylation activity and functions exclusively as a *trans*-editing factor. This alternation is facilitated by the modularity of ThrRSs and the relative independence of the aminoacylation and editing domains (16,22,23). According to these results, *apo*-T1 could be added to an increasing list of *trans*-editing factors that include stand-alone proteins like AlaX, YbaK, ProXP or archaeal ThrRS-ed (18–20,54) and complete aaRSs like PheRS and ProRS, which are able to compete with elongation factor Tu for binding misacylated tRNAs and rapidly hydrolyze them (55).

It has been shown that distinct proofreading mechanism may be used to prevent the production of a particular misacylated tRNA in different cell types (49) or cell compartments (45,50). In this work we show that in the same cell alternative proofreading mechanisms are used depending on the cell status. In zinc sufficiency, when T1 is in the *holo* state, Ser-tRNA^Thr^ produced by T1 could be edited *in cis* before being released (16). By contrast in zinc deficiency, when T1 is in the *apo* state, Ser-tRNA^Thr^ molecules produced by T2 would be captured by T1 and edited *in trans*. Also in the context of heterodimers, editing by *apo*-T1 would be exerted *in trans* as the distance between the active site of T2 and the editing site of *apo*-T1 is too long to permit translocation of the tRNA acceptor arm. It is also probable that *holo*-T1 edit released Ser-tRNA^Thr^ *in trans*, as shown for other class II aaRSs like PheRS or ProRS (55). The *in vivo* relative contribution of *cis*- and *trans*-editing of Ser-tRNA^Thr^ remains to be determined but it could be anticipated that in zinc deficiency the contribution of *trans*-editing is likely essential given the low *cis*-editing activity of T2.

Functional switches are a common theme for aaRSs, especially in complex organisms. Some aaRSs alternate the aminoacylation function with a variety of roles that may be totally unrelated to their canonical function. Function alternation may occur with no enzyme modifications but in general it follows proteolytic processing, post-translational modifications, dissociation from a complex, alternative splicing or combinations of these (14). In the case described here the functional switch of T1 is determined by a change in its oligomeric state caused by allosteric interaction with zinc.

### Oligomerization plasticity as a means to generate aaRS variability beyond the genetic repertoire

Trans-oligomerization of duplicated aaRSs is a possibility thus far ignored in the literature of these enzymes and to our knowledge, *apo*-T1-T2 is the only heterodimeric ThrRS characterized to date. However, the existence of duplicated genes encoding ThrRS in many organisms, including the cytoplasm of human cells (7), raises the possibility of the operation in other species of heterodimeric ThrRSs.

Out of ca. 26,000 cases of duplicated aaRS in organisms of the three domains of life only a handful have been empirically characterized. In these few cases, duplicated aaRSs were shown to have evolved distinct expression profiles (8,56,57) substrate specificity (58–63) susceptibility to antibiotics (9,64,65) or thermal stability (66). In general, it is proposed that the asymmetric properties of duplicated proteins provide a selective advantage, a better fitness or a higher robustness to the host organism (67). In our system, the cell not only benefits from possessing two ThrRSs with dissimilar properties but also from the possibility of generating combinations of these by oligomerization. Thus, this oligomerization plasticity of duplicated aaRSs is a means to generate aaRS variants exceeding the genetic repertoire, i.e. three or six dimeric variants can be assembled out of duplicated or triplicated aaRSs, respectively. Most importantly, we observe that the composition of dimeric ThrRSs best fits the requirements of the cell in each situation. For instance, we observe that the heterodimers that are formed *in vivo* under zinc deficiency are composed of T2 subunits that provide low-zinc-resistant synthetic activity and *apo*-T1 subunits that provide editing activity *in trans*. Therefore, dynamic trans-oligomerization of duplicated aaRSs is a *bona fide* adaptive mechanism operating through the assemblage of the oligomers that best fit the physiologic status of the cell.

Since many aaRSs are oligomeric, including all class II and some class I aaRSs, we propose that the controlled oligomerization of duplicated aaRSs is an adaptive mechanism potentially widespread among the plethora of duplicated or triplicated oligomeric aaRSs in Nature, and possibly among other duplicated oligomeric proteins. Oligomers would have the combined functional capabilities of the subunits, as observed for T1-T2, or would display novel functionality emerging from the interaction of distinct subunits. While in our system the control of oligomerization is exerted by allosteric interaction with zinc, in other systems it could be mediated by other allosteric effectors, by the control of gene expression or by post-translational modifications.

We speculate that heterooligomerization could also occur in eukaryotes with no duplicated aaRSs given the possibility of generating multiple polypeptides by alternative splicing. That would be an extraordinary means to create aaRSs variability that may be tuned to the cell status, including the cell cycle phase or the differentiation stage of the cell.

## Supplementary Material

SUPPLEMENTARY DATA
