# Supplementary material for: Trans-oligomerization of duplicated aminoacyl-tRNA synthetases maintains genetic code fidelity under stress
Source: Nucleic Acids Res. 2015 Oct 12;43(20):9905–17. doi: 10.1093/nar/gkv1020 (PMC4787780; doi:10.1093/nar/gkv1020)
Supplement: SUPPLEMENTARY DATA [file supp_43_20_9905__index.html]

Trans-oligomerization of duplicated aminoacyl-tRNA synthetases maintains genetic code fidelity under stress — SUPPLEMENTARY DATA 

# Trans-oligomerization of duplicated aminoacyl-tRNA synthetases maintains genetic code fidelity under stress

## SUPPLEMENTARY DATA

- SUPPLEMENTARY DATA
- SUPPLEMENTARY DATA
