# Supplementary material for: Trans-oligomerization of duplicated aminoacyl-tRNA synthetases maintains genetic code fidelity under stress
Source: Nucleic Acids Res. 2015 Oct 12;43(20):9905–17. doi: 10.1093/nar/gkv1020 (PMC4787780; doi:10.1093/nar/gkv1020)
Supplement: SUPPLEMENTARY DATA [file supp_gkv1020_nar-02261-z-2015-File011.pdf]

Table S1. Plasmids used in this work

Name	Description	Source
pTrc99a	*lac*-promoter-based expression plasmid	http://www.addgene.org/vector-database/4402/#
pCMN8	Derivative plasmid of pRL278 (Elhai & Wolk, 1988) carrying the *thrS2* gene interrupted by the insertion at the HindIII site of the C.S3 cassette conferring resistance to streptomycin and spectinomycin	This work
pCMN10	Derivative plasmid of pRL278 (Elhai & Wolk, 1988) carrying the *thrS1* gene interrupted by the insertion at the EcoRI site of the C.S3 cassette conferring resistance to streptomycin and spectinomycin	This work
pCMN15	pTrc99A bearing a NcoI/XhoI fragment containing the *thrS1* gene(*alr0335*) from *Anabaena* with a 5' His-tag	This work
pCMN16	pTrc99A bearing a NcoI/XhoI fragment containing the *thrS2* gene(*all4723*) from *Anabaena* with a 5' His-tag	This work
pCMN17	pTrc99A bearing a EcoRI/SalI fragment containing the *thrS* gene from *E. coli.*	This work
pCMN18	Plasmid based on pCMN15 bearing the *trnT*(CGU) gene from *Anabaena* downstream *thrS1*	This work
pCMN19	Plasmid based on pCMN16 bearing the *trnT*(CGU) gene from *Anabaena* downstream *thrS2*	This work
pCMN28b	Overexpression plasmid based on the comercial vector pET28b (InvitroGen) where the HisTag coding sequence has been substituted by a Strep-Tag II coding sequence using primers Strep-Tag1F and 1R	Napolitano et al. (2012)
pCMN20	Plasmid yielding a NdeI/XhoI fragment bearing gene *thrS2* (*all4723*) from *Anabaena* PCC 7120 cloned into pCMN28b.	This work
pCMN21	Plasmid yielding a NdeI/XhoI fragment bearing gene *thrS1* (*alr0335*) from *Anabaena* PCC 7120 cloned into pCMN28b.	This work
pCMN30	Plasmid derived of pET28b bearing the ORFs of *thrS2* and *thrS1* fused to sequences encoding a His tag and a StrepTag-II, respectively	This work
pCMN31	Plasmid derived of the pRL278 conjugative vector of *Anabaena* containing an insert encompassing the 3'-end of the *all4724* gene and a sequence encoding a StrepTag-II sequence fused to the 5' end of *thrS2*.	This work
pCMN50	Plasmid yielding a NdeI/XhoI fragment bearing gene *trnT*(CGU) from *Anabaena* PCC 7120 cloned into pCMN28b.	This work
pCi20	Plasmid derived of pET28b bearing the ORFs of *thrS2* and *thrS1*-SYY fused to sequences encoding a His tag and a StrepTag-II, respectively	This work
pCMA49	Plasmid derived from pCMN21 overexpressing T1-S	This work
pCMA53	Plasmid derived from pCMN21 overexpressing T1-SY	This work
pCMA54	Plasmid derived from pCMN21 overexpressing T1-SYY	This work
pCMA47	Plasmid derived from pCMN21 overexpressing T1Δ*b*	This work
pCMA48	Plasmid derived from pCMN21 overexpressing T1Δ*c*	This work
pCMA60	Plasmid derived from pCMN21 overexpressing T1Δ*bc*	This work
pCMA68	Plasmid derived from pCMN20 overexpressing T2ΔN	This work
pCMA67	Plasmid derived from pCMN20 overexpressing T2Δ*a*	This work

Table S2. Oligonucleotides used in this work

Name	Sequence (5'-3')	Purpose
E.coli-ThrS-F	GCAAATAAGAATTCAAAATGCCTG	Amplification of the *thrS* gene from *E. coli*. Also adds EcoRI/SalI restriction sites for cloning into the pTrc99a vector
E.coli-ThrS-R	GACCTGTCGACCGAACTTCCTGGG	
A7120 -CGU-1F	GTTCAGGATCCAAACGCTTTTTGG	Amplification of the *trnT* gene from *Anabaena* and introduction of BamHI/HindIII restriction sites for cloning into the pCMN28b vector
A7120 -CGU-1R	CTTTAGATAAGCTTTATAAATAGCCT CT	
A7120 -CGU-2F	CGGCTCCATTTTCATAAAATCATAT	Partially overlapping primers designed to add a CCA sequence at the 3' end of the *trnT* gene encoding $tRNA^{Thr}$(CGU)
A7120- CGU-2R	GAAAATGGAGCCGATGATGGGATTTG	
Strep-Tag 1F	CATGGCAAGCTGGAGCCACCCGCAGT TCGAAAAGGGTGCACTTGAAGTCCTC TTTCAGGGACCCCA	Overlapping primers that carry the sequence of the Strep-TagII plus NcoI/NdeI restriction sites to replace the HisTag portion of the pET28b.
Strep-Tag 1R	TATGGGGTCCCTGAAAGAGGACTTCA AGTGCACCCTTTTCGAACTGCGGGTG GCTCCAGCTTGC	
A7120-THRS1-2F	CACTTTGTTACGCAGCACCT	Q-PCR
A7120-THRS1-2R	TTTGCCAGTGTCCTGAAGTC	Q-PCR
A7120-THRS2-2F	AAGCCAACCTGCCAATAATC	Q-PCR
A7120-THRS2-3R	AAGTAGCGGGTGATGGTTTC	Q-PCR
A7120-RNPB-1F	TTGGTAACAAACGTCCCAGA	Q-PCR
A7120-RNPB-1R	TAAGCCGGGTTCTGTTCTCT	Q-PCR
7120TRNAGLU-1F	GGCGATTGGATATTTCTTAG	Used to generate a probe to detect $tRNA^{Glu}$ by Northern
7120TRNAGLU-1R	CCTGAAGGGATTTAAGTTAGTAT	
RS1_ser_FW	CAATGAATAGCCCCTTCCAC	Mutation of Cys308 of T1 to Ser
RS1_ser_RV	GTGGAAGGGGCTATTCATTG	
A7120-Thrs1-3F	ATGATTCTTACTTATTCGTGACCCCA	Mutation of His359 of T1 to Tyr
A7120-Thrs1-3R	CACGAATAAGTAAGAATCATCAACAG	
A7120-Thrs1-4F	TGATGATTTACCGTGCGCCTTTC	Mutation of His488 of T1 to Tyr
A7120-Thrs1-4R	CGCACGGTAAATCATCACGGGAC	
thrRS1_indel2_FW	CAAGGCTTTGTCCTCAAGC	Divergent primers for amplification of the complete sequence of pCMN21 but the portion comprising indel *b*
thrRS1_indel2_RV	TTCATCCTCAGCCATCAGG	
thrRS1_indel3_FW	AAGTACATCGGTTCTGATGAAG	Divergent primers for amplification of the complete sequence of pCMN21 but the portion comprising indel *c*
thrRS1_indel3_RV	TGGGTCACGGAAACTGAGT	
RS2_deltaindel1_FW	AACTATTGGTGGGACTTGTGC	Divergent primers for amplification of the complete sequence of pCMN20 but the portion comprising indel *a*
RS2_deltaindel1_RV	ACCAATAAAGTAGCGGGTGAT	
RS2_domN2_FW	CACAGCTCATATGACAAAGGCAG	Divergent primers for amplification of the complete sequence of pCMN20 but the portion comprising the N1-N2 domain
RS2_domN2_RV	GGAACTGACCATATGAATAAAT	

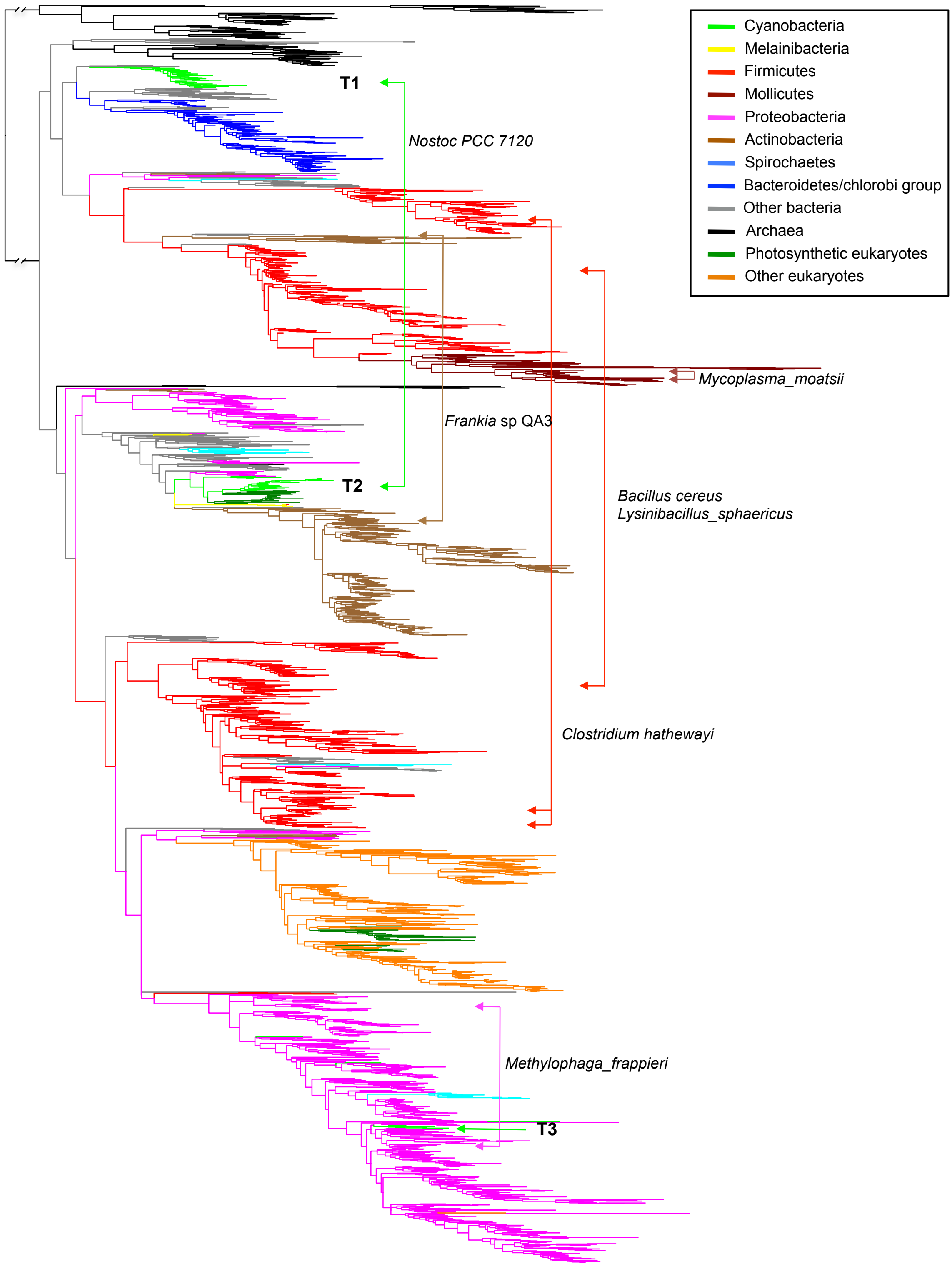

Figure S1. Phylogeny of ThrRSs. Branches are coloured according to the code indicated in the box. Connected arrows mark the two ThrRS copies of some representative organisms following the same colour code as branches. Distant connected arrows (as in *Bacillus cereus*) indicate that ThrRSs originated from ancient gene duplication or lateral gene transfer (LGT) from distant species. Proximal connected arrows (as in *Mycoplasma moatsii*) indicate that ThrRSs originated from recent gene duplication or LGT from related species. The position of cyanobacterial T1, T2 is shown. T3 is a ThrRS acquired by horizontal gene transfer by cyanobacteria of the *Prochlorococcus* genus.

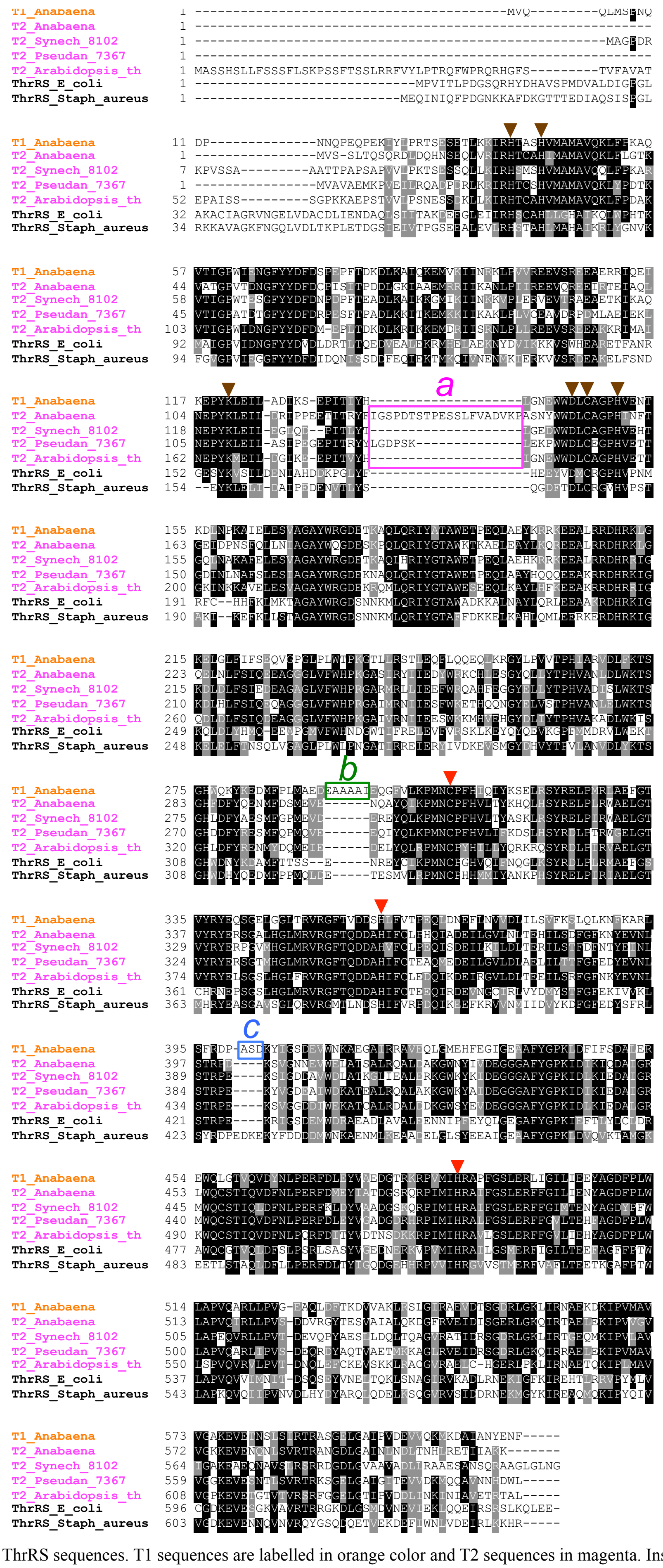

Figure S2. Alignment of ThrRS sequences. T1 sequences are labelled in orange color and T2 sequences in magenta. Insertions *a, b* and *c* are boxed. Red and brown arrowpoints indicate zinc coordination residues and amino acids involved in editing, respectively.

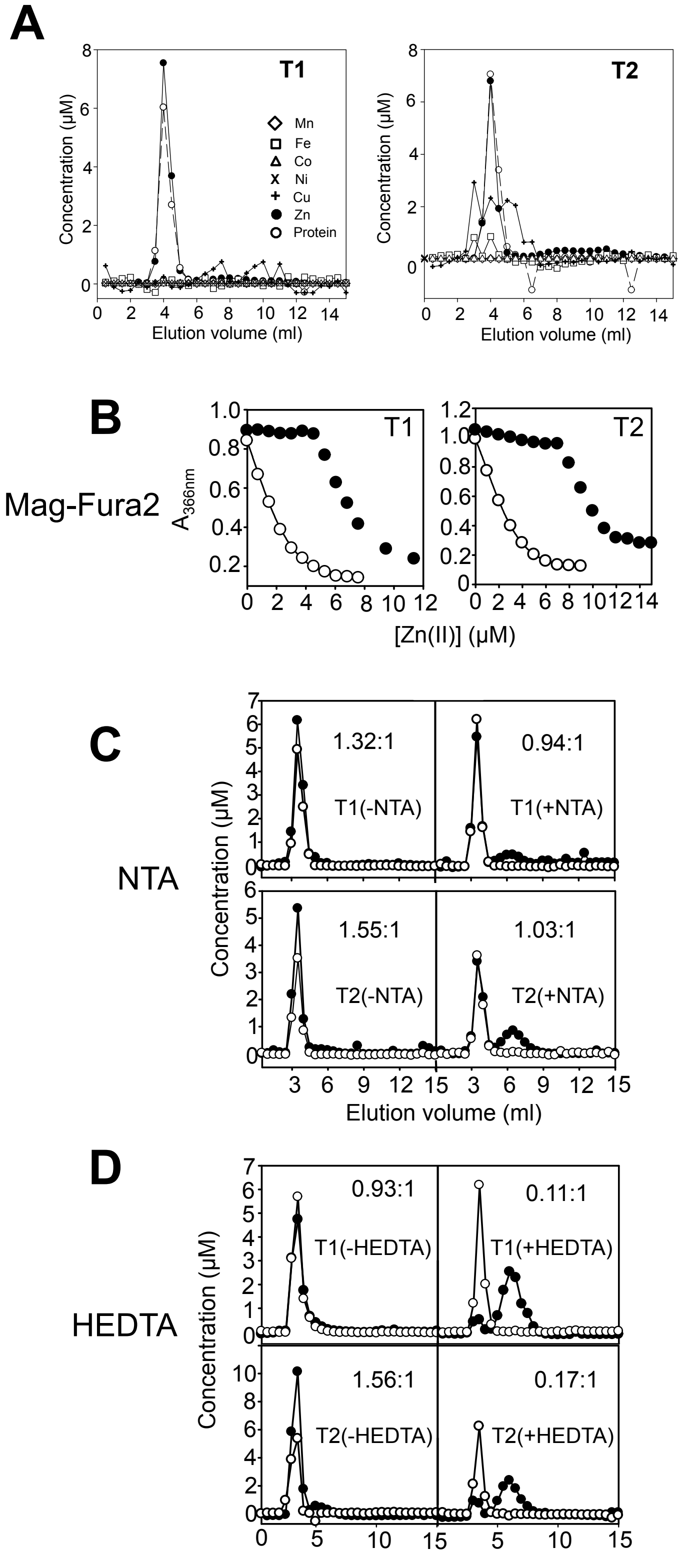

Figure S3. (A) Metal analysis of recombinant T1 and T2. Pure preparations of each proteins were subjected to gel filtration using buffer previously demetallated by two successive filtrations by Chelex-100 resin. Protein and metal content of fractions were determined by Bradford assays and ICP-MS. (B) Metal competition for Zn(II) during titration of 5 µM Mag-Fura-2 in the presence (closed symbols) or absence (open symbols) of 5 µM T1 (left) or T2 (right) recombinant proteins. (C and D) Competition of 10 µM of T1 (top panels) and T2 (bottom panels) for Zn(II) in the presence (right panels) or absence (left panels) of 20 µM NTA (C) or 20 µM HEDTA (D). Samples were processed by gel filtration as described in A. Numbers indicate the Zn(II):protein ratio of the peak fractions. Open and solid symbols indicate the protein and metal concentration, respectively.

Comments on Fig. S3

These experiments indicate that both T1 and T2 possess a zinc affinity intermediate to NTA and HEDTA, as at pH 7.5 Mag-Fura-2 has a zinc binding affinity of 2×10^{-8} M (1), NTA of 3.74×10^{-9} M and HEDTA of 5.96×10^{-13} M. Intriguingly three separate experiments (Fig S3B right panel, Fig S3C lower-left panel and Fig S3D lower-left panel) suggest that the T2 isoform may have a higher zinc binding stoichiometry than the T1 isoform, three rather than two atoms per dimer. Interestingly, a stoichiometry above two atoms per dimer has also been observed for *E. coli* ThrRS incubated in the presence of 5 mM $ZnCl_2$ but not for a mutant lacking the N-terminal domain, suggesting that the extra metal binding site could be in this domain (2,3). This was further supported by the inhibitory action of zinc or nickel ions on the oxidation of the cysteine residue in the editing site (4). Also consistent with this is the observation of X-ray scattering in the crystal of ThrRS of *Staphylococcus aureus* proposed to result from a metal ion bound to the editing site (5). The physiological relevance of this elevated zinc binding stoichiometry of T2 remains to be elucidated.

1. Simons, T.J. (1993) Measurement of free Zn2+ ion concentration with the fluorescent probe mag-fura-2 (furaptra). J Biochem Biophys Methods, 27, 25-37.
2. Dock-Bregeon, A., Sankaranarayanan, R., Romby, P., Caillet, J., Springer, M., Rees, B., Francklyn, C.S., Ehresmann, C. and Moras, D. (2000) Transfer RNA-mediated editing in threonyl-tRNA synthetase. The class II solution to the double discrimination problem. Cell, 103, 877-884.
3. Dock-Bregeon, A.C., Rees, B., Torres-Larios, A., Bey, G., Caillet, J. and Moras, D. (2004) Achieving error-free translation; the mechanism of proofreading of threonyl-tRNA synthetase at atomic resolution. Mol Cell, 16, 375-386.
4. Wu, J., Fan, Y. and Ling, J. (2014) Mechanism of oxidant-induced mistranslation by threonyl-tRNA synthetase. Nucleic Acids Res, 42, 6523-6531.
5. Torres-Larios, A., Sankaranarayanan, R., Rees, B., Dock-Bregeon, A.C. and Moras, D. (2003) Conformational movements and cooperativity upon amino acid, ATP and tRNA binding in threonyl-tRNA synthetase. J Mol Biol, 331, 201-211.

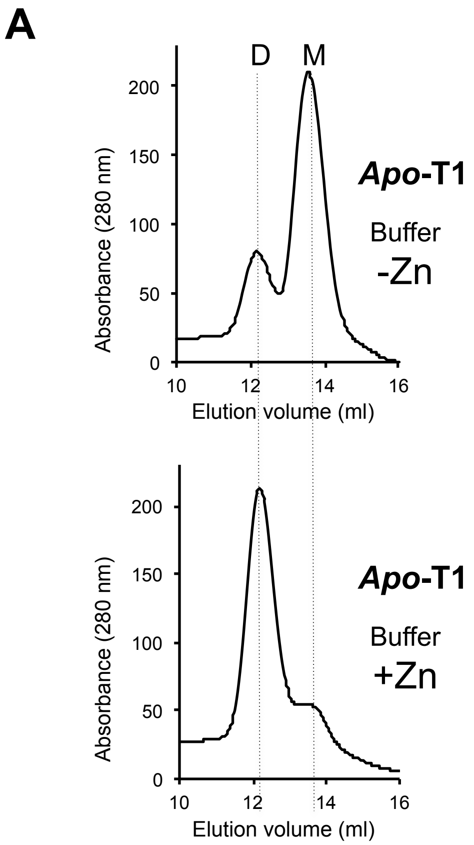

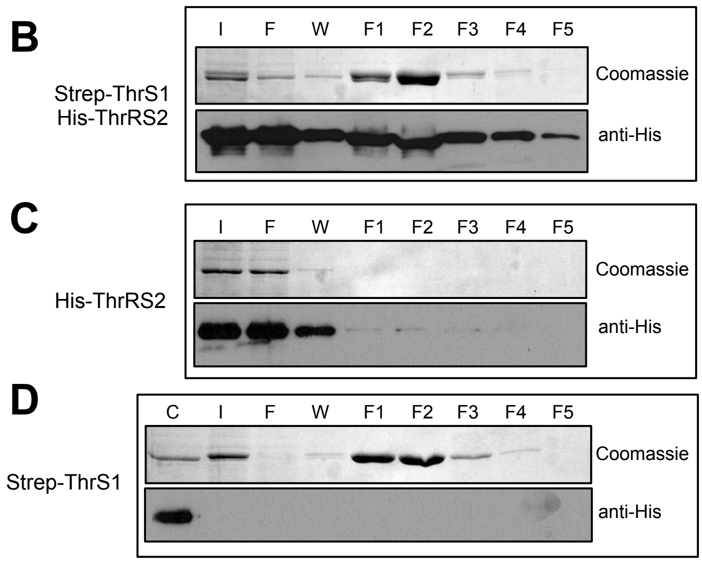

Fig. S4. Homodimerization and heterodimerization by T1. (A) Dissociation of T1 is a reversible process. Pure preparations of T1 protein were incubated for 24 h in the presence of 5 mM EDTA and subjected to gel filtration in buffer containing 150 mM NaCl, 100 mM Tris-HCl pH 7.5, 1 mM DTT, supplemented with 5 mM EDTA (top panel) or 5 mM $ZnSO_4$ (bottom panel). "D" and "M" indicate the elution volume corresponding to the size of a dimer (150 KDa) or a monomer (75 KDa), respectively. (B, C and D) Extracts form *E. coli* expressing the proteins indicated at the left were subjected to streptavidine affinity chromatography, and fractions were resolved by SDS-PAGE and stained with coomassie or transferred to membranes and incubated with anti-hexahistidine antibodies, as indicated at the right. Letters "I, F, W and F1-F5" indicated respectively input, flow through, wash, and elution fractions 1 to 5. Letter "C" on top of the bottom panel indicate a positive control for the western.

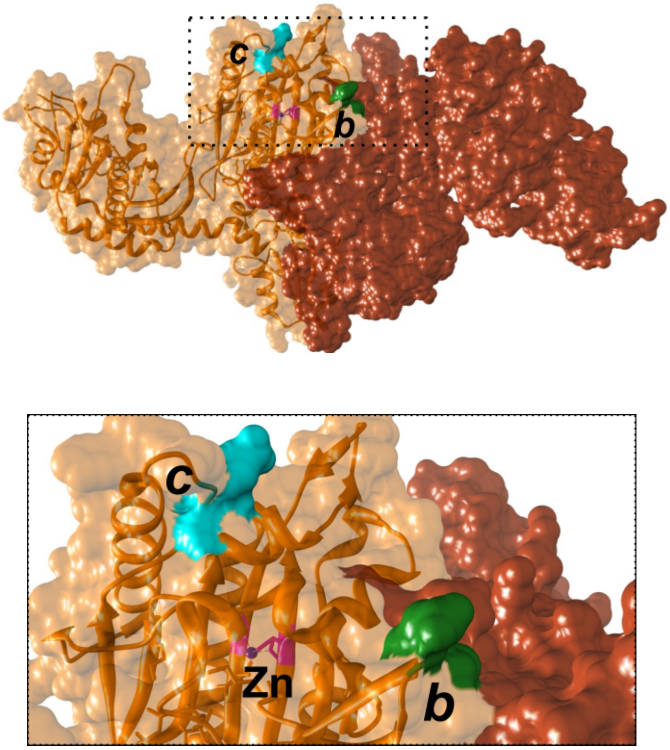

Fig. S5. Localization of insertions *b* and *c* in the three dimensional structure of T1.
The structure of the *Staphylococcus aureus* ThrRS (PBD id: 1NYR) was used to highlight residues E_{325} and T_{326} flanking the insertion point of insert *b* in *Anabaena* T1 (in green) and residues D_{429}, K_{430} and E_{431} that align with residues of insert *c* in *Anabaena* T1. Subunits are depicted in different orange tones. Zn is shown in dark blue and Zn-binding residues in magenta.
